# Clinical value of combined serum CA125, NSE and 24-hour urine VMA for the prediction of recurrence in children with neuroblastoma

**DOI:** 10.1186/s13052-023-01508-6

**Published:** 2023-08-24

**Authors:** Jinmin Li, Zilong Qi, Mo Chen, Jiachen Wang, Xiangyang Liu

**Affiliations:** 1 Pediatric Surgery Department, Cangzhou Central Hospital, Children’s Hospital District, Intersection of Guangrong Road, Fuyang South Avenue, 061000 Cangzhou, Hebei China; 2 Disinfection & Supply Department, Cangzhou Central Hospital, No. 16 Xinhua West Road, 061000 Cangzhou, Hebei China

**Keywords:** Neuroblastoma, CA125, NSE, VMA, Children

## Abstract

**Background:**

In this study, we intend to retrospectively analyze the clinical data of postoperative neuroblastoma children, including the results of follow-up examinations and laboratory tests, to explore the clinical value of combined serum Carbohydrate antigen 125 (CA125), neuron-specific enolase (NSE) and 24-hour urine vanillylmandelic acid (VMA) levels at baseline for the prediction of recurrence in children with neuroblastoma.

**Methods:**

265 children with neuroblastoma were successfully followed up, including 163 cases without recurrence (non-recurrence group) and 102 cases with recurrence (recurrence group). The levels of 24-hour urine VMA were determined using spectrophotometric methods. Additionally, the serum levels of CA125 and NSE were measured using electrochemiluminescence immunoassay.

**Results:**

The serum CA125, NSE and 24-hour urine VMA levels were significantly higher in the recurrence group than in the non-recurrence group. It demonstrated a significant positive correlation between the levels of serum CA125, NSE, and 24-hour urine VMA in all children with neuroblastoma. All children in stage IV of neuroblastoma had the highest level of serum CA125, NSE and 24-hour urine VMA and vice versa. The combined CA125, NSE and VMA had significantly better sensitivity and specificity than an individual marker.

**Conclusions:**

Combined serum CA125, NSE and 24-hour urine VMA had the potential to predict neuroblastoma recurrence more effectively.

## Background

Neuroblastoma is one of the most common malignant tumors in children, which occurs in the developing sympathetic nervous system [1]. Neuroblastoma accounts for approximately 8% of all pediatric cancers [2]. The presentation of the neuroblastoma at primary stage can vary depending on its location and size. Common symptoms associated with the primary tumor include abdominal pain or distension, a palpable mass, urinary or bowel dysfunction [3]. Patients in metastasis stage mainly present with limb pain, claudication or pathological fractures, with liver and skin lesions [4]. Neuroblastoma is insidious and difficult to detect in the early stages. Moreover, the probability of remission and recurrence after resection surgery is much higher in children with neuroblastoma compared to other malignant solid tumors, with up to 50% of patients following a dismal outcome [5].

In recent years, due to improvements in chemotherapy regimens and combination therapies, the remission rate of neuroblastoma after treatment has increased significantly [6]. Nevertheless, recurrence and metastasis after remission still require salvage therapy. Therefore, effective follow-up of neuroblastoma children in complete remission after treatment is essential. If recurrence and metastasis can be accurately and sensitively monitored at an early stage to predict the prognosis, targeted treatment can be given at an early stage, undoubtedly prolonging the survival time and improving the quality of life of the children.

Tumor markers can assist in reflecting the process of tumor development and predicting and evaluating the treatment prognosis. Due to their low invasiveness, simple operation, and reproducibility, they are widely used in clinical practice. Testing tumor markers is one of the main follow-up tools for most tumors. Status of MYCN oncogene has been recognized as one of risk factors for neuroblastoma. Moreover, numerous studies have been conducted on several tumor markers related to neuroblastoma, including neuron-specific enolase (NSE) [7, 8], vanillylmandelic acid (VMA) [9, 10], lactate dehydrogenase (LDH) [11], homovanillic acid (HVA) [12, 13] and ferritin [14, 15]. Higher levels of NSE in the serum of pediatric patients with neuroblastoma often indicate a more advanced disease stage and may be indicative of a poor prognosis [16]. VMA is a metabolite of catecholamines, which are chemicals produced by neuroblastoma tumors. The measurement of urinary VMA serves as a valuable diagnostic and monitoring tool in pediatric neuroblastoma [17, 18]. Studies have shown that elevated serum ferritin levels at diagnosis or during follow-up are correlated with a higher risk of relapse, metastasis, and overall survival rates of neuroblastoma [14]. Serum HVA levels of neuroblastoma patients were 15–30 times higher than that of the normal control group [12]. The serum LDH is an essential indicator of the systemic tumor load, where the higher LDH value indicates the worse the child’s prognosis [11].

Based on our previous study [19], where we found that serum carbohydrate antigen 125 (CA125) of children in the effective treatment group were significantly lower than those in the treatment-ineffective group, we decided to include CA125 as a marker in this study. CA125 is commonly associated with certain types of cancers, particularly ovarian cancer [20]. However, studies have shown that neuroblastoma can also influence CA125 levels, leading to an increase in serum concentration [21, 22]. It is believed that neuroblastoma cells may produce or release substances that can trigger the increase in CA125 levels.

These markers are entensively used in the initial evaluation of neuroblastoma and are considered valuable tools for follow-up. However, the positive rate of individual serum tumor markers has been low [23]. In this study, we intend to retrospectively analyze the clinical data of postoperative neuroblastoma children, including the results of follow-up examinations and laboratory tests, to explore the clinical value of combined serum CA125, NSE and 24-hour urine VMA levels at baseline for the prediction of recurrence in children with neuroblastoma.

## Methods and materials

### Study participants

This is a monocentric study. We used the commonly employed five-year survival rate as the follow-up cutoff date to assess tumor recurrence or non-recurrence. In this study, 265 children with neuroblastoma were successfully followed up, including 163 cases without recurrence (non-recurrence group) and 102 cases with recurrence (recurrence group). The study was approved by the Ethical Committee of Cangzhou Central Hospital. Written consent was obtained from the participant’s parents or guardians.

The stages of all patients were assigned according to the International Neuroblastoma Staging System (INSS) [24]: Stage I, the tumor is limited to the primary tissue or organ; Stage II, the tumor spreads to the vicinity of the primary tissue or organ, but does not go beyond the midline, with ipsilateral regional lymph node metastasis; Stage III, the tumor goes beyond the midline, with bilateral lymph node metastasis; Stage IV, distant metastasis to bones, soft tissues or lymph nodes.

### Inclusion criteria

Patients with (1) neuroblastoma confirmed by pathology after surgery; (2) meeting the diagnostic criteria for neuroblastoma as guidelines of Pediatric Oncologic Surgery [25]; (3) age less than 14 years at the time of preoperative diagnosis; (4) defined as neuroblastoma recurrence or metastasis according to guidelines of The International Neuroblastoma Response Classification (INRC) [26] during the first five years of follow-up after initial surgery.

### Exclusion criteria

Patients with (1) severe heart, liver or kidney diseases, coagulation disorders, cognitive dysfunction, neurological, autoimmune or haematological diseases; (2) a combination of other malignant tumors; (3) died of non-neuroblastoma recurrence or metastasis within five years of follow-up after the first surgery.

### Measurement

One week before the test, all children during the diagnosis were prohibited from consuming food containing vanillins, such as chocolate, bananas and lemons. The 24-hour urine was collected, and 5 to 10 mL of concentrated hydrochloric acid was added for preservation. The total amount of urine was recorded, and finally, 30–50 mL was selected and sent for testing. When children were admitted to the hospital, 5 ml of fasting venous blood was collected and centrifuged at 3000 r/min for 10 min. The serum was collected for testing. The levels of 24-hour urine VMA were determined by spectrophotometric methods using a high-performance liquid chromatograph. Serum LDH, ferritin and HVA levels were measured using electrochemiluminescence immunoassay (using Roche E411 electrochemiluminescence immunoassay analyzer). Additionally, the serum CA125, MYCN and NSE levels were also measured using the same electrochemiluminescence immunoassay method.

### Surgery procedure

Before surgery, the patients were classified into risk groups based on risk classification system developed by Children’s Oncology Group [3].

Surgery and chemotherapy plan for intermediate-risk group: Scheduled surgery is performed before or during chemotherapy (approximately 4 cycles), followed by postoperative chemotherapy until partial response (PR) is achieved for 4 cycles, with a maximum of 8 cycles in total. If necessary, a second surgery may be performed. After completion of chemotherapy, maintenance treatment is administered with 13-cis-retinoic acid (13-cis-RA) at a dose of 160 mg/m2, given for 14 days per month, for a total of 6 months.

Surgery and chemotherapy plan for high-risk group: The treatment consists of three phases, including the induction phase (including chemotherapy and surgery), consolidation phase (sequential transplantation and radiotherapy targeting primary tumor and residual metastatic sites), and maintenance therapy after consolidation (immunotherapy and 13-cis-RA). Specifically, after two cycles of chemotherapy, autologous bone marrow stem cells are collected, followed by two additional cycles of chemotherapy and scheduled surgery. After surgery, two more cycles of chemotherapy are administered, with a maximum of 6 cycles in total. After completion of standard chemotherapy, sequential autologous bone marrow transplantation (ABMT) and radiotherapy to the tumor bed (sequential ABMT is recommended, with radiotherapy performed between two ABMT procedures) are conducted. Immunotherapy in combination with 13-cis-RA treatment follows.

Radiotherapy: High-risk patients over 18 months of age require radiotherapy to the primary site and metastatic lesions. Palliative radiotherapy is suitable for patients whose condition has progressed and poses a threat to vital organs, or when the tumor has rapidly increased in size and affects the respiratory system or causes digestive system obstruction. It is also applicable for patients experiencing symptoms such as pain, compression, or bleeding from bone and soft tissue metastases.

### Statistical analysis

Statistical Package for the Social Sciences (SPSS) software version 19.0 was used for data sorting and analysis. The measurement data were presented as mean ± standard deviation (SD). Mann-Whitney test or Fisher’s exact test, or Chi-square test was performed to find significant differences between the groups. The prediction value was evaluated by non-parametric receiver operating characteristic (ROC) analyses. The area under the curve (AUC) showed diagnostic accuracy. P < 0.05 was considered statistically significant.

## Results

### Basic characteristics

The basic information and clinical indexes of 265 children with pediatric neuroblastoma between recurrence and non-recurrence groups during five years follow-ups were collected and shown in Table 1. After comparing the basic information at baseline of the non-recurrence and recurrence group, there were no significant differences in age and gender (p > 0.05). According to INSS, 42 children (25.8%) were diagnosed at stage I, 56 (34.3%) at stage II, 38 (23.3%) at stage III and 27 (16.6%) at stage IV in the non-recurrence group. There were 15 children (14.7%) diagnosed at stage I, 21 (20.6%) at stage II, 39 (38.2%) at stage III and 27 (26.5%) at stage IV in the recurrence group. There were significant differences in the INSS stage between the two groups (p = 0.001). In terms of treatment, all patients in both the non-recurrence and recurrence groups underwent surgery and chemotherapy, with 163 cases (100%) in each group. Radiotherapy was administered to 59 cases (36.2%) in the non-recurrence group and 48 cases (47.1%) in the recurrence group. The majority of recurrences occur locally (71.6%), followed by bone metastasis (8.8%), bone marrow metastasis (6.9%), pelvic metastasis (5.9%), CNS metastasis (3.9%), and pulmonary metastasis (2.9%). The median time to recurrence was 14 months. There were no significant differences observed in baseline serum NSE, CA125, and 24-hour urine VMA levels among the different types of recurrence.

**Table 1:** Clinical characteristics at baseline of the children with pediatric neuroblastoma between recurrence and non-recurrence group during 5 years follow-up

Variables	Study group		p
	Non-recurrence (n = 163)	Recurrence (n = 102)	
Gender			
Male	79 (48.5%)	57 (55.9%)	0.258
Female	84 (51.5%)	45 (44.1%)	
Age			
< 18 months	76 (46.6%)	38 (37.3%)	0.161
≥ 18 months	87 (53.4%)	64 (62.7%)	
INSS stage			
I	42 (25.8%)	15 (14.7%)	0.001
II	56 (34.3%)	21 (20.6%)	
III	38 (23.3%)	39 (38.2%)	
IV	27 (16.6%)	27 (26.5%)	
Primary site			
Mediastinum	32 (19.6%)	28 (27.5%)	0.177
Adrenal	79 (48.5%)	36 (35.3%)	
Abdomen	39 (23.9%)	30 (29.4%)	
Others	13 (8.0%)	8 (7.8%)	
MYCN			
Normal	112 (68.7%)	59 (57.8%)	0.086
Amplified	51 (31.3%)	43 (42.2%)	
Treatment			
Surgery	163 (100%)	102 (100%)	1
Chemotherapy	163 (100%)	102 (100%)	1
Radiotherapy	59 (36.2%)	48 (47.1%)	0.095
Serum LDH (U/L)	628.12 ± 193.43	674.64 ± 237.51	0.108
Serum HVA (µmol/L)	56.16 ± 20.45	72.28 ± 24.02	0.084
Serum ferritin (ng/mL)	148.32 ± 47.48	269.81 ± 62.97	0.006
Serum CA125 (U/ml)	55.84 ± 26.06	82.20 ± 37.01	< 0.001
Serum NSE (ng/mL)	51.02 ± 25.54	75.06 ± 31.51	< 0.001
24-hour urine VMA (µmol/L)	67.28 ± 30.46	111.22 ± 49.48	< 0.001
Recurrence pattern			
Local relapse	-	73 (71.6%)	-
CNS metastasis		4 (3.9%)	
Bone metastasis		9 (8.8%)	
Bone marrow metastasis		7 (6.9%)	
Pulmonary metastasis		3 (2.9%)	
Pelvic metastasis		6 (5.9%)	

Values presented are mean ± SD or n (percentage). The comparisons of data between the Non-recurrence and Recurrence groups were done by Mann Whitney test or Fisher’s exact test or Chi-square test

INSS: International Neuroblastoma Staging System. LDH: Lactate dehydrogenase. CA125: carbohydrate antigen 125. NSE: Neuron specific enolase. VMA: Vanillylmandelic acid. HVA: Homovanillic acid. CNS: Central nerve system

In addition, amplification rate of MYCN between the two groups were compared. Based on the results, there were no significant differences in amplification rate of MYCN between the two groups (p > 0.05). The serum LDH, HVA, ferritin, CA125, NSE and urine VMA of the two groups were evaluated and compared. The results suggest that there are significant differences in serum CA125, NSE, and 24-hour urine VMA levels between the non-recurrence and recurrence groups (p < 0.01), while serum LDH and HVA levels did not show significant differences (p = 0.108, 0.084, respectively).

### Serum CA125, NSE and 24-hour urine VMA levels were correlated with neuroblastoma recurrence

The serum CA125, NSE, and 24-hour urine VMA levels were evaluated in both the recurrence and non-recurrence groups during a five-year follow-up period, as shown in Table 1. The purpose was to investigate the association between CA125, NSE, and VMA levels and neuroblastoma recurrence.

**Fig. 1:**
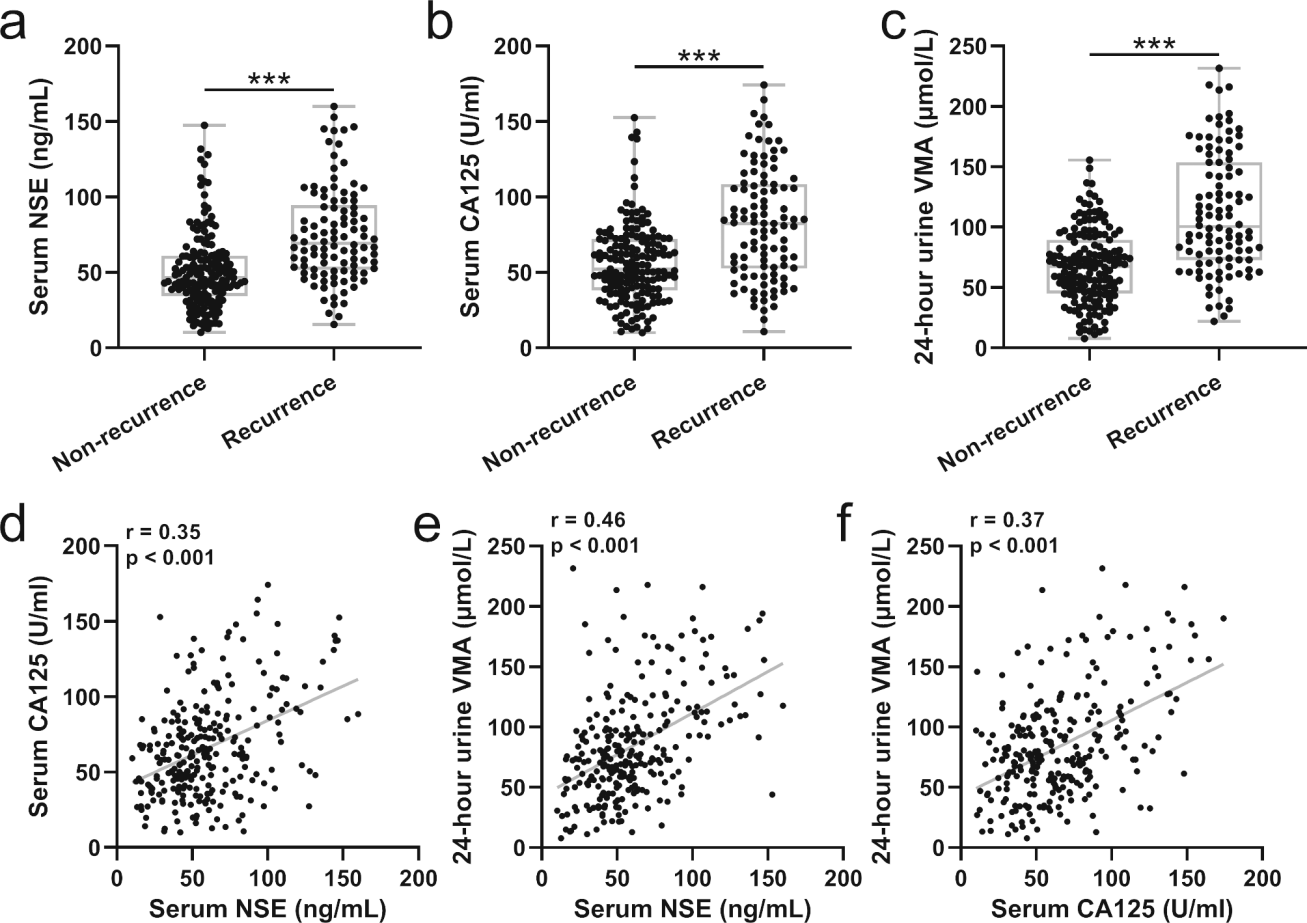
Comparisons of serum NSE (**a**), CA125 (**b**) and 24-hour urine VMA (**c**) at baseline of the children with pediatric neuroblastoma between recurrence (n = 102) and non-recurrence (n = 163) group during 5 years follow-up. Box plots with all data points were used to present the data. *** p < 0.001. Mann Whitney test. Spearman’s correlation analysis was used to measure the correlations between serum NSE and CA125 (**d**), serum NSE and 24-hour urine VMA (**e**), serum CA125 and 24-hour urine VMA (f) in children with pediatric neuroblastoma. n = 265

Figure 1a-c; Table 1 demonstrate that children with neuroblastoma recurrence had significantly higher levels of serum CA125, NSE, and 24-hour urine VMA compared to those without neuroblastoma recurrence (p < 0.05). Additionally, Spearman’s correlation analysis was conducted to assess the relationship between serum CA125, NSE, and 24-hour urine VMA levels among all children with neuroblastoma, as depicted in Fig. 1d-f. The analysis revealed a significant positive correlation between the levels of serum CA125, NSE, and 24-hour urine VMA (p < 0.05).

### Serum CA125, NSE and 24-hour urine VMA levels were elevated with the severity of neuroblastoma

**Fig. 2:**
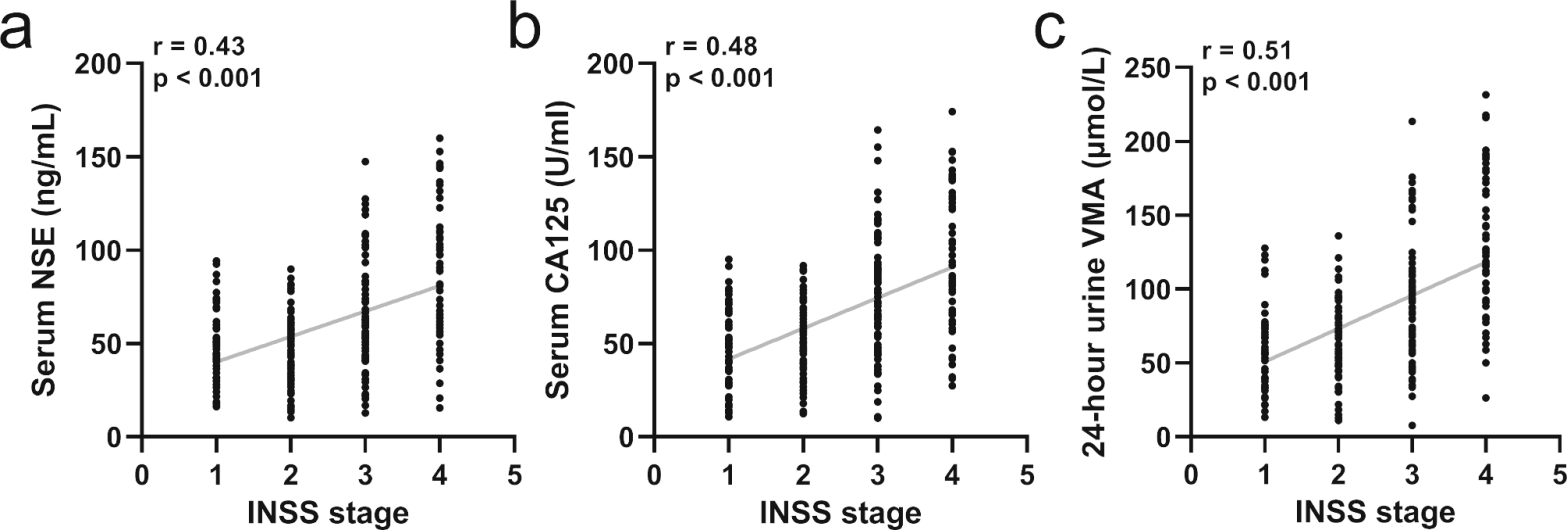
Spearman’s correlation analysis was used to measure the correlations between INSS stage with serum NSE (**a**), CA125 (**b**) and 24-hour urine VMA (**c**) in children with pediatric neuroblastoma. n = 265

We examined the relationship between the INSS stage and the levels of serum CA125, NSE, and 24-hour urine VMA. The results demonstrated a significant positive correlation between the INSS stages and the levels of serum CA125, NSE, and 24-hour urine VMA in all children, as depicted in Fig. 2a-c.

Specifically, children diagnosed with stage IV neuroblastoma generally exhibited the highest levels of serum CA125, NSE, and 24-hour urine VMA, while those with lower stages showed comparatively lower levels. This finding suggests that the severity of neuroblastoma is associated with elevated levels of serum CA125, NSE, and 24-hour urine VMA.

**Combined measurement of serum CA125, NSE and 24-hour urine VMA had the potential to predict neuroblastoma recurrence more effectively**.

**Fig. 3:**
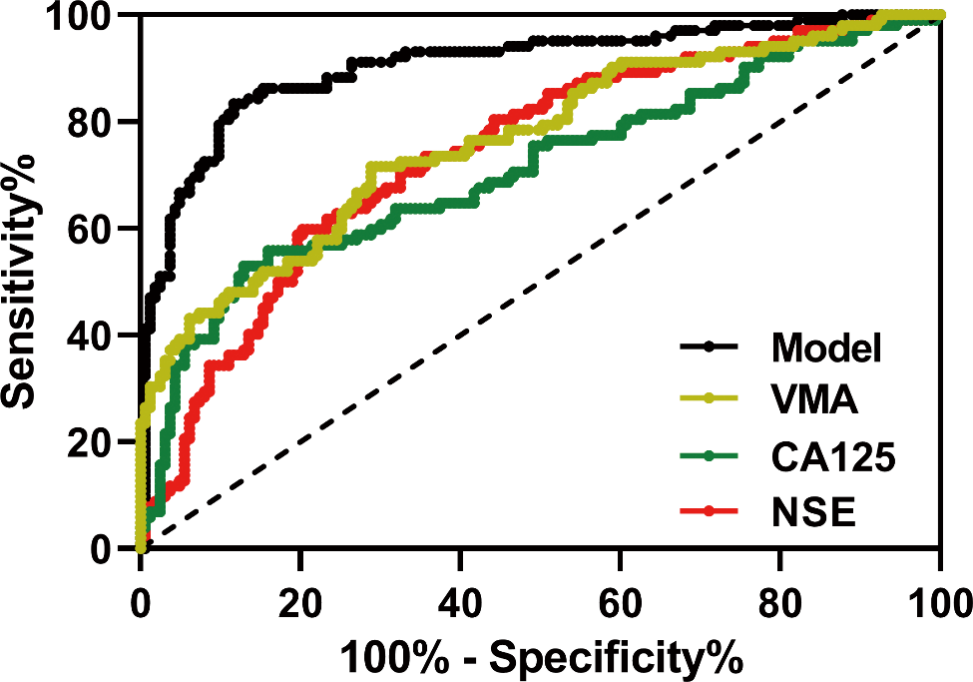
ROC analysis of serum NSE, CA125, 24-hour urine VMA and their combination model for the predictive value of recurrence in children with neuroblastoma

ROC analysis was processed to confirm the predictive value of CA125, NSE, VMA and their combined assay for postoperative neuroblastoma recurrence. The serum CA125, NSE, 24-hour urine VMA and the combined assay generated the area under the curves (AUC) of 0.71 (0.64 to 0.77), 0.74 (0.68 to 0.80), 0.76 (0.70 to 0.82) and 0.91 (0.87 to 0.95), respectively (p < 0.001) (Fig. 3; Table 2). The results demonstrated that CA125, NSE and VMA have predictive value for neuroblastoma recurrence in children. Moreover, the combined measurement of serum CA125, NSE and 24 h-urine VMA demonstrated significantly improved sensitivity and specificity compared to using an individual marker. Therefore, it suggests that combined measurement of serum CA125, NSE, and 24-hour urine VMA had the potential to more effectively predict neuroblastoma recurrence in children with neuroblastoma.

**Table 2:** Predictive values in ROC analysis

	Cut-off value	AUC (95% CI)	p	Sensitivity (%)	Specificity (%)	Youden index
Serum CA125	79.93	0.71 (0.64 to 0.77)	< 0.001	52.94	87.12	0.40
Serum NSE	63.69	0.74 (0.68 to 0.80)	< 0.001	59.80	79.75	0.39
24-hour urine VMA	79.95	0.76 (0.70 to 0.82)	< 0.001	71.57	71.17	0.43
Model^*^	-	0.91 (0.87 to 0.95)	< 0.001	83.33	88.34	0.72

CI: confidence interval

^*^Logit (Model) = 0.032 * NSE + 0.027 * CA125 + 0.030 * VMA

## Discussion

Neuroblastoma is one of the malignant tumors that poses a significant threat to children’s health. The five-year survival rate of high-risk neuroblastoma patients is generally less than 50% [27] and the survival ratio of children with neuroblastoma recurrence is also very low [28]. Great attention should be paid to the management of a complex pathology in the pediatric age [29]. Diagnosis of neuroblastoma according to the INSS criteria usually requires a tissue biopsy of the tumor lesion. However, during clinical practice, many children with neuroblastoma have already been in the advanced stage with huge tumors and extremely poor physical condition. In this condition, biopsy surgery is risky and may result in complications, such as massive intraoperative and postoperative bleeding, tumor rupture and abdominal infection. Therefore, it is crucial to accurately and noninvasively identify children with neuroblastoma recurrence at an early stage to facilitate appropriate clinical therapies. Finding biomarkers to assist tumor diagnosis and evaluate the prognosis of treatment is a popular area of cancer research. In our study, we investigated the clinical correlation between levels of serum CA125, NSE and urine VMA on the recurrence and metastasis of neuroblastoma. Our aim was to identify a monitoring method with high specificity and sensitivity to predict neuroblastoma recurrence at an early stage.

CA125 is a carbohydrate antigen from the coeliothel, epithelium of the female genitourinary tract, mesothelial cells, the serous membranes and mucosal cells of the stomach and colon [30]. Elevated serum concentrations of CA125 were observed and used as biomarkers for screening in several malignancies, such as ovarian cancer, endometrial cancer, colon cancer, breast cancer and lung cancer [31–33]. Serum CA125 also showed a significant increase in adult neuroblastoma [21]. Based on our previous study, where we found that serum CA125 of children in the effective treatment group were significantly lower than those in the treatment-ineffective group, we decided to include CA125 as a marker in this study [19]. NSE is a glycolytic enzyme found in neuronal tissues and tumors [22]. NSE is a sensitive tumor marker in neuroblastoma, which correlates with tumor burden and stages [8, 19, 34]. High serum NSE levels often indicate a poor prognosis of pediatric neuroblastoma [16]. However, NSE is not specific to neuroblastoma. Elevated serum NSE could also be a valuable indicator for other neuroendocrine cancer [35]. VMA is excreted in urine and is a significant product of norepinephrine and epinephrine metabolism. Previous studies demonstrated that VMA could be a marker of neuroblastoma [35, 36]. In particularly, it has been suggested that the ratio of VMA and HVA have prognostic significance in neuroblastoma [36, 37]. Urinary VMA levels in children with neuroblastoma have shown predictive value for therapy effectiveness [19], and urinary VMA has also been correlated with the prognosis of localized tumors in pelvic, thoracic and neck [36].

CA125, NSE and VMA are all proven makers of neuroblastoma. However, all of them have other correlated diseases or tumors. In our study, serum CA125, NSE and 24-hour urine VMA were considered to combine in predicting neuroblastoma recurrence. The results showed that serum CA125, NSE and 24-hour urine VMA levels were significantly higher in the children with neuroblastoma recurrence than in the non-recurrence group. Meanwhile, children in stage IV of neuroblastoma had the highest level of serum CA125, NSE and 24-hour urine VMA and vice versa. Furthermore, serum CA125, NSE and 24-hour urine VMA levels were elevated with the severity of neuroblastoma. Furthermore, the result of ROC analysis demonstrated that the combined CA125, NSE and VMA had significantly better sensitivity and specificity than individual markers. Therefore, combined serum CA125, NSE, and 24-hour urine VMA had the potential to predict neuroblastoma recurrence more effectively in children with neuroblastoma.

## Conclusion

In conclusion, serum CA125, NSE and 24-hour urine VMA levels were correlated with neuroblastoma and elevated with the severity of neuroblastoma. In addition, combined serum CA125, NSE and 24-hour urine VMA had the potential to predict neuroblastoma recurrence more effectively.

## Data Availability

All data generated or analyzed during this study are included in this published article.

## List of abbreviations

NSE: neuron-specific enolase
VMA: vanillylmandelic acid
CA125: carbohydrate antigen 125
